# Nectar Guide Patterns on Developmentally Homologous Regions of the Subtribe Ligeriinae (Gesneriaceae)

**DOI:** 10.3389/fpls.2021.650836

**Published:** 2021-04-12

**Authors:** Hao-Chun Hsu, Yan-Fu Kuo

**Affiliations:** Department of Biomechatronics Engineering, National Taiwan University, Taipei City, Taiwan

**Keywords:** image processing, geometric transformation, Gesneriaceae, Ligeriinae, nectar guide pattern, petal vasculature, region of interest, homologous region

## Abstract

Homology is a crucial concept that should be considered while conducting a comparative analysis between organisms. In particular, in the subtribe Ligeriinae, the nectar guide pattern is associated with high diversity in petal shapes and sizes. This largely limits researchers to exclusively examining the interspecific variation in nectar guide patterns on the developmentally homologous region. Thus, to solve this problem, we proposed an approach for defining a homologous region of interest (ROI) that aligns the petal image between specimens based on petal contours and vasculatures. We identified petal contours and vasculatures from the fresh petal image and its histological image through image processing. The homologous ROI was subsequently obtained by applying geometric transformation to the contour and vasculature. The qualification and quantification of nectar guide patterns were subsequently performed based on the homologous ROI. Four patterning modes, namely vascular, random, distal, and proximal, were defined for the qualitative analysis of nectar guide patterns. In the quantitative analysis, principal component (PC) analysis was applied to homologous ROIs, and the PC score of each specimen served as the trait values of nectar guide patterns. The results of the two analyses coincided, and both showed significant associations between nectar guide patterns and pollination types. The proximal mode (corresponding to PC1) and distal mode (corresponding to PC2) together showed the strongest association with pollination types. Species exhibiting the hummingbird and bee pollination types tended to recruit the distal and proximal modes, respectively. Our study conducted a comparative analysis of nectar guide patterns on the developmentally homologous region and provided a comprehensive view of the variation in the nectar guide patterns of Ligeriinae.

## Introduction

Nectar guide patterns, which are observed as contrasting pigmentation on flower petals, have attracted biologists’ interest for centuries. The diversity of these patterns is considered a key morphological characteristic that facilitates the plant–pollinator interaction and angiosperm diversification (Penny, 1983; Endress, 1996; van der Kooi et al., 2019). Several studies have examined variations in nectar guide patterns (Yoshioka et al., 2004; Lootens et al., 2007; Hsu et al., 2018; Hung et al., 2020). In addition to these nectar guide patterns, petal shape and size exhibit considerable diversity. In particular, shape differences lead to misunderstanding of the distribution of pigmented areas and overestimation of variation in nectar guide patterns. To prevent this problem, considering the developmentally homologous region of petals is essential. This study proposed an approach that considers the developmentally homologous region of petals to examine the variation in nectar guide patterns.

Petal vasculature is an ideal object for considering the developmentally homologous regions of petals. The petal, the second whorl of a flower, is considered a homologous structure across most core eudicots (Endress, 1996; Ronse De Craene, 2008). Moreover, the petal recruits many fundamental genetic bases of lateral organ development, including vascular differentiation (Walcher-Chevillet and Kramer, 2016; Basile et al., 2017). In some taxa, the vasculature is a frequently used characteristic for examining the variation in petals (Gómez and Perfectti, 2010; Wang et al., 2020). These studies have acquired spatial information not only from contours but also from the vasculature. For petals with different shapes, the vasculature, together with lobe contours, can serve as the anchor to study the distribution of pigmented areas based on the developmentally homologous region.

The inclusion of developmental homologous region in the definition of a region of interest (ROI) may facilitate the examination of nectar guide patterns in different petal shapes. In image processing analysis, defining the ROI is a frequently used approach to specify the study object in an image. Many studies have applied ROI to quantify petal color patterns among species or varieties (Yoshioka et al., 2004; Lootens et al., 2007; Hsu et al., 2018; Hung et al., 2020). Although these studies have successfully evaluated the variation in nectar guide patterns, ROIs obtained from different petal shapes may not be homologous. For example, Hung et al. (2020) defined an ROI on the basis of mechanical traits such as petal widths and lengths. In other words, the position of ROIs fluctuates between species, and even between specimens, in different petal shapes. Whibley et al. (2006) used a consistent corolla shape obtained through geometric transformation to analyze interspecific variations in flower colors. Therefore, the developmentally homologous region of the nectar guide patterns in different petal shapes can be considered. In this study, we propose a homologous ROI that aligns the contour and vasculature of different petal shapes to analyze nectar guide patterns.

**FIGURE 1:**
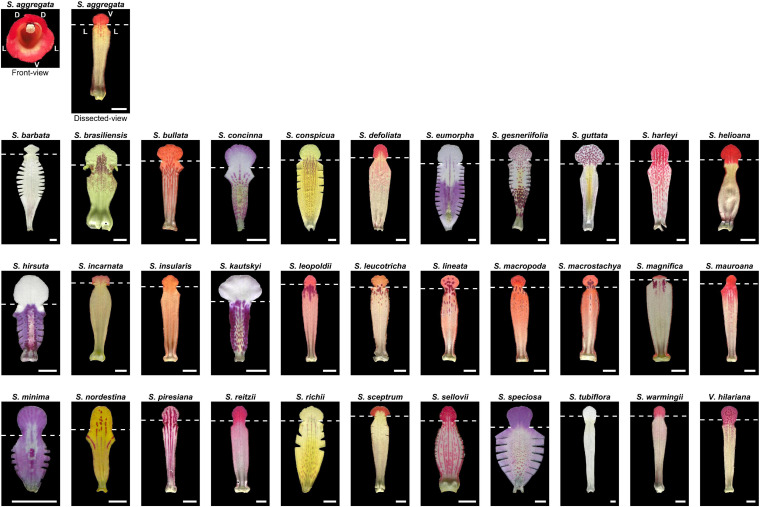
Ventral petals of 34 Ligeriinae species. The white dashed line denotes the location of the lobe–tube connected rim. Scale bar: 0.5 cm.

The subtribe Ligeriinae is an ideal system for implementing the image-based approach to examine nectar guide patterns. The subtribe Ligeriinae was recognized as a monophyletic group in the family Gesneriaceae, and composed of the species from three genera, *Sinningia*, *Vanhouttea*, and *Paliavana*. A study reported considerable variations in corolla shapes and nectar guide patterns and the pollination types of most species in the subtribe Ligeriinae (Perret et al., 2007). The developmental anatomy of flowers is consistent throughout the subtribe. The wild varieties of all species have a zygomorphic corolla with two dorsal, two lateral, and one ventral petal (top right of Figure 1). The vasculature of each petal contains one middle vein and two side veins (Wang et al., 2020). In addition, the ventral petal has a showy nectar guide pattern with color gradients, dotted stripes, and spot clusters (Figure 1). Because the ventral petal serves as the landing and contacting platform for visiting pollinators, it has a simple and flat structure. This provides an innate advantage for capturing high-quality images of petals under optimal imaging conditions. By defining a homologous ROI in the image, we considered developmentally homologous regions in the analysis of nectar guide patterns.

In this study, we collected images of fresh ventral petals and histological images of 34 Ligeriinae species. The homologous ROI of petals was defined and obtained from the fresh petal image and the histological image through image processing. On the basis of the homologous ROI, we aimed to (1) qualify and quantify nectar guide patterns and (2) extend our understanding of the association between nectar guide patterns and pollination types in the subtribe Ligeriinae.

## Materials and Methods

### Sampling and Growth Conditions

The sampled species were selected such that they covered a range of corolla morphologies (shape, size, and color) and pollination types in the subtribe Ligeriinae (Table 1). Plant materials used for the qualification and quantification of nectar guide patterns were sampled from the greenhouse of the Dr. Cecilia Koo Botanic Conservation Center (KBCC, Pingtung, Taiwan). The growth conditions were as follows: natural lighting with 60% net shading, humidity of 70–80%, and temperature of 22–30°C. Specimen collection was conducted from 2017 to 2019. Specimens were collected at the floral developmental stage between the anther and stigma anthesis. Specimens of the same species were collected in the same flowering season. In total, 454 specimens of 34 species were collected.

**TABLE 1:** Species list and number of specimens, pollination types, and accession numbers of the sampled individuals.

	**Number of specimen**	**Pollination type^a^**	**KBCC accession number**
*Sinningia aggregata*	26	Hummingbird	K039091, K039092, K039093, K039297
*Sinningia barbata*	3	Bee	K039105
*Sinningia brasiliensis*	17	Bat	K023999, K024028, K024030, K024040, K024074
*Sinningia bullata*	42	*To be determined*	K023430, K023431, K023443, K023448, K037111
*Sinningia concinna*	10	Bee	K039117
*Sinningia conspicua*	5	Bee	K039121
*Sinningia defoliata*	3	Hummingbird	K023265, K039125
*Sinningia eumorpha*	36	Bee	K039133
*Sinningia gesneriifolia*	9	*To be determined*	K039220, K039221
*Sinningia guttata*	3	Bee	K039134
*Sinningia harleyi*	7	Hummingbird	K039135
*Sinningia helioana*	6	*To be determined*	K039222
*Sinningia hirsute*	4	Bee	K039136
*Sinningia incarnata*	3	Hummingbird	K039137
*Sinningia insularis*	28	Hummingbird	K039142, K039145
*Sinningia kautskyi*	9	Bee	K039146, K039147
*Sinningia leopoldii*	9	Hummingbird	K039148, K039150
*Sinningia leucotricha*	6	Hummingbird	K039157
*Sinningia lineata*	43	Hummingbird	K039159, K039162, K039163
*Sinningia macropoda*	19	Hummingbird	K023363, K023405, K023413, K039164
*Sinningia macrostachya*	9	Hummingbird	K023944, K039165
*Sinningia magnifica*	4	Hummingbird	K039166
*Sinningia mauroana*	10	Hummingbird	K024149, K024154, K024169
*Sinningia minima*	4	*To be determined*	S82P101^*b*^
*Sinningia nordestina*	29	Hummingbird	K039168
*Sinningia piresiana*	9	Hummingbird	K023312, K023333
*Sinningia reitzii*	15	Hummingbird	K039173
*Sinningia richii*	5	Bee	K039176
*Sinningia sceptrum*	10	Hummingbird	K039178, K039182
*Sinningia sellovii*	21	Hummingbird	K023224, K039184, K039185
*Sinningia speciosa*	11	Bee	K039190
*Sinningia tubiflora*	4	Moth	K039191
*Sinningia warmingii*	14	Hummingbird	K039206, K039207
*Vanhouttea hilariana*	21	Hummingbird	K039218

*^*a*^Pollination type was referenced from the study by Perret et al. (2007). ^*b*^Plant material held by HC Hsu.*

### Fresh Petal Image Acquisition

After a flower was harvested, the corolla was dissected along the middle vein of the two lateral petals. From the ventral half of the corolla, the remaining lateral lobes were removed, and a necessary cut was made at the remaining lateral tube region to properly flatten the petal (Figure 1). An image of the adaxial surface of the petal was captured using a flatbed color scanner (Perfection V370, Epson, Nagano, Japan). The resolution ranged from 600 to 9,600 dpi. During scanning, the petal was flattened at the scanning stage and covered with a black cloth. The detailed procedure was reported by Hung et al. (2020). Subsequently, the scanned fresh petal was carefully moved to a fixative solution.

### Histological Image Acquisition

The scanned fresh petal was fixed with 0.5% glycerol (v/v) in a 70% ethanol solution under a 600-mmHg vacuum until the tissue was completely infiltrated. The ethanol-fixed petal was subsequently transferred to the scanner and covered with a light emitting diode light board (A4 ultra-thin portable light box, LitEnergy, Dallas, TX, United States) under a color temperature of 9,000 K and a light source of 4,000 lux. The resolution was the same as that used in fresh petal image acquisition.

### Extraction of the Homologous ROI

**FIGURE 2:**
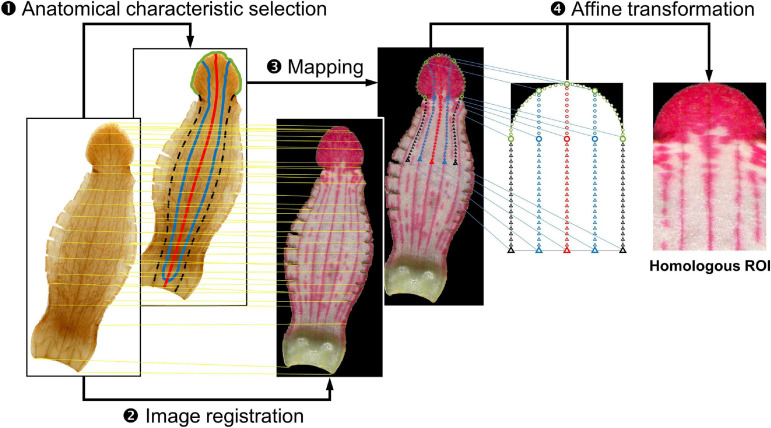
Homologous region of interest (ROI) extraction procedure. In the histological image, the green line denotes the lobe contour, the red line denotes the track of the middle vein, the blue line denotes the track of the side vein, and the black dotted line denotes the tube–tube boundary. The yellow line connecting the histological image and fresh petal image denotes landmarks that were found in both images and used for image registration. The blue line connecting the fresh petal image and the frame of the homologous ROI denotes the source point and destination point pair.

The homologous ROI was first defined as the frame combining a half-circle and a rectangle corresponding to the lobe and tube regions in the fresh petal image, respectively (Figure 2). The homologous ROI corresponded to the entire lobe and one-third of the tube at the distal side. Anatomical characteristics, namely the lobe contour (green line), the tracks of the middle vein (red line) and two side veins (blue line), and the tube–tube boundary between the ventral and lateral regions (black dotted line), were acquired to align the lobe contour and vasculature of specimens in different petal shapes and sizes.

Homologous ROI extraction was performed in four steps: anatomical characteristic selection, image registration, mapping, and affine transformation. The anatomical characteristic selection was performed on the histological image. Anatomical characteristics included the lobe contour, the tracks of the middle and side veins, and the tube–tube boundary. The lobe contour was automatically identified using the Gaussian-mixture-model-based segmentation algorithm (Nasios and Bors, 2006). The tracks of the middle vein and two side veins were manually identified using the cursor. The tube–tube boundary between the ventral and lateral regions was determined by examining the middle line between the track of the ventral side vein and the track of the neighboring lateral side vein. Subsequently, image registration was performed by superimposing the control points of histological images over the corresponding control points of fresh petal images (Gonzalez and Woods, 2006). The control points were landmarks found in both the images, such as the tips on the serrated lobe edge and the cutting trace of the lateral tube region. The identified anatomical characteristics were then mapped to the registered fresh petal image. Subsequently, 161 source points were arranged on the mapped characteristics (empty circles and triangles on the fresh petal image in Figure 2). In addition, the frame of the homologous ROI was arranged with 161 destination points. The source point and the corresponding destination point were paired. Finally, the homologous ROI was obtained through affine transformation (Gonzalez and Woods, 2006). The aforementioned procedures were performed using MATLAB (MathWorks, Natick, MA, United States).

### Qualification of Nectar Guide Patterns

**FIGURE 3:**
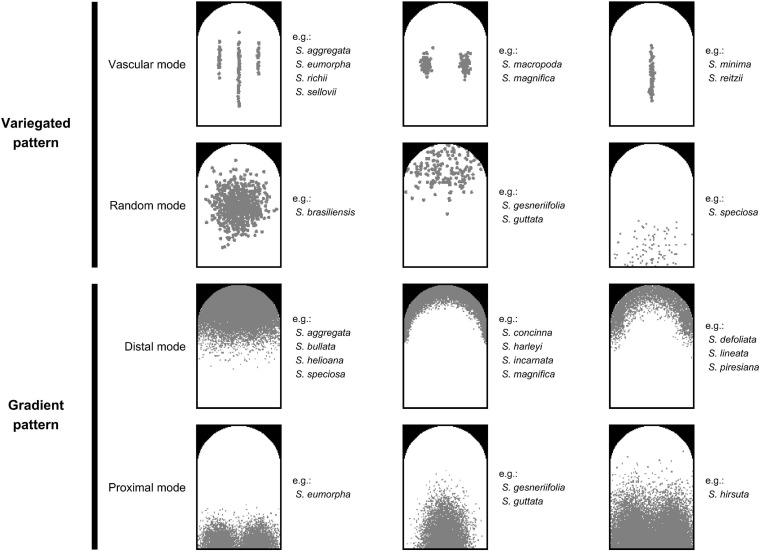
Illustrations of four patterning modes. The gray region denotes the location of the pigmented area.

On the basis of the observation of homologous ROIs, four patterning modes were defined to qualify the nectar guide patterns. In addition to our previous study in which we classified the pattern of petal colors in Ligeriinae (Hung et al., 2019), we also incorporate the observation on the developmental serial to support the definition of patterning modes (Supplementary Figure 1). The variegated pattern was defined as pigmentation initiating at the center of the petal, and the gradient pattern was defined as pigmentation initiating at the distal or proximal end of the petal. In this study, two patterning modes of each pattern were identified (Figure 3). In the variegated pattern, the pigmentation overlaying the vasculature was defined as the vascular mode (Figure 3, first row), and the distribution of pigmentation that does not follow the vasculature was defined as the random mode (Figure 3, second row). In the gradient pattern, the pigmentation initiating from the distal end was defined as the distal mode (Figure 3, third row), and pigmentation initiating from the proximal end was defined as the proximal mode (Figure 3, fourth row).

The association between the qualitative nectar guide pattern and pollination type was evaluated using the χ^2^ test of independence. Cross-tabulation was performed to match the patterning modes of the variegated and gradient patterns with pollination type. The level of significance was set to 0.05.

### Quantification of Nectar Guide Patterns

Nectar guide patterns were quantified by performing principal component analysis (PCA). The homologous ROI of each specimen was first converted from red–green–blue to grayscale and transformed from a two-dimensional image to a one-dimensional vector. The collection of one-dimensional vectors was then subjected to PCA. In PCA, variations in nectar guide patterns were decomposed into a series of principal components (PCs), which corresponded to an essential characteristic of nectar guide patterns. Inverse PCA was applied to PC scores to visualize variations in nectar guide patterns. In addition, stepwise multiple regression was applied to identify PCs that contributed to pollination types.

The association between quantitative nectar guide patterns and pollination types was evaluated by calculating the logarithm of the odds (LOD) score and by performing a permutation test. The LOD score in each PC is the logarithm of the ratio of the squared deviation of the PC score to the sum of the within-type squared deviation of the PC score (Broman and Sen, 2009). The equation of LOD score is as,

(1) $$\mathrm{LOD}\,\mathrm{score}=\,\frac{n}{2}\,\log_{10}\frac{\sum_{i}{\left(y_{i}-\bar{y}\right)}^{2}}{\sum_{i}{\left(y_{i}-{\hat{y}}_{p\,i}\right)}^{2}}$$

where the *n* is the sample size, the *y*_*i*_ is the PC score of specimen *i*, the $\bar{y}$ is the mean PC score of all specimens, the ${\hat{y}}_{p\,i}$ is the mean PC score of the pollination type of specimen *i*. The permutation test was then performed to evaluate the statistical significance of the LOD score. In the test, pairs of PC scores and their corresponding pollination types were shuffled 10,000 times. In each shuffle, the LOD score was calculated according to the shuffled pairs. The collection of LOD scores obtained from 10,000 shuffles served as the null distribution of the permutation test. The *P*-value for the test was subsequently calculated as the possibility that the null distribution exceeds the LOD score calculated from the original PC score–pollination type pair. A lower *P*-value indicated a statistically significant association between the quantified nectar guide pattern and pollination type.

## Results

**FIGURE 4:**
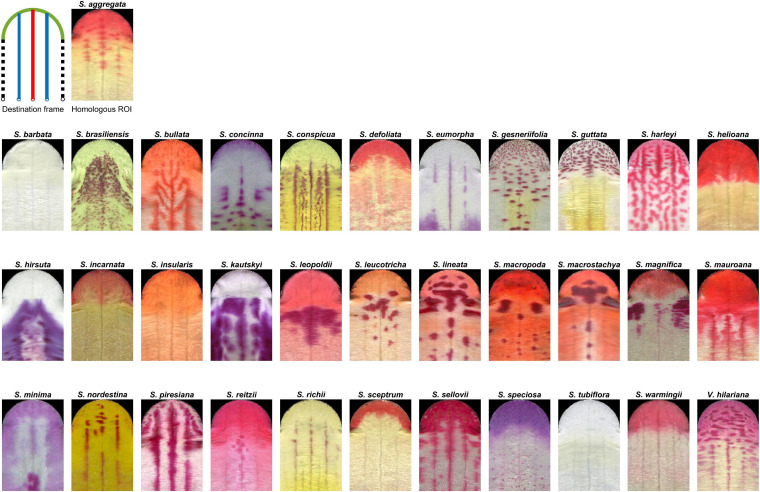
Homologous ROIs of 34 Ligeriinae species. The legend for line colors is identical to that in Figure 2.

The homologous ROI unified the lobe contour and vasculature from different species. Figure 4 shows the homologous ROI for each species. All homologous ROIs were subsequently used for the qualification and quantification of nectar guide patterns.

### Qualitative Nectar Guide Patterns and Their Association With Pollination Types

Table 2 lists the qualitative nectar guide patterns of 34 Ligeriinae species. Four patterning modes, namely the vascular and random modes of the variegated pattern and the distal and proximal modes of the gradient pattern, were determined on the basis of the location of the pigmented area in the homologous ROIs. Some species, such as *Sinningia brasiliensis* and *Sinningia conspicua*, acquired only a single patterning mode. Most of the species with complex patterns acquired two patterning modes. Moreover, the vascular and random modes were mutually exclusive in the variegated pattern, and the distal and proximal modes were mutually exclusive in the gradient pattern.

**TABLE 2:** Qualitative nectar guide patterns of 34 Ligeriinae species.

	**Variegated pattern (Vascular mode vs. Random mode)**	**Gradient pattern (Distal mode vs. Proximal mode)**
*Sinningia aggregata*	V	D
*Sinningia barbata*	–	–
*Sinningia brasiliensis*	R	–
*Sinningia bullata*	V	D
*Sinningia concinna*	V	D
*Sinningia conspicua*	V	–
*Sinningia defoliata*	V	D
*Sinningia eumorpha*	V	P
*Sinningia gesneriifolia*	R	P
*Sinningia guttata*	R	P
*Sinningia harleyi*	V	D
*Sinningia helioana*	–	D
*Sinningia hirsuta*	R	P
*Sinningia incarnata*	–	D
*Sinningia insularis*	–	D
*Sinningia kautskyi*	V	P
*Sinningia leopoldii*	V	D
*Sinningia leucotricha*	V	D
*Sinningia lineata*	V	D
*Sinningia macropoda*	V	D
*Sinningia macrostachya*	V	–
*Sinningia magnifica*	V	D
*Sinningia mauroana*	V	D
*Sinningia minima*	V	D
*Sinningia nordestina*	V	–
*Sinningia piresiana*	V	D
*Sinningia reitzii*	V	D
*Sinningia richii*	V	–
*Sinningia sceptrum*	–	D
*Sinningia sellovii*	V	D
*Sinningia speciosa*	R	D
*Sinningia tubiflora*	–	–
*Sinningia warmingii*	–	D
*Vanhouttea hilariana*	V	D

The patterning modes of the variegated and gradient patterns showed strong associations with pollination types (Table 3, χ^2^ = 16.70, *P* = 1.05 × 10^–2^; Table 4, χ^2^ = 22.75, *P* = 8.85 × 10^–4^). No pigmentation was considered a patterning mode (None in Tables 3, 4). The species exhibiting the hummingbird pollination type tended to recruit the vascular mode of the variegated pattern and the distal mode of the gradient pattern.

**TABLE 3:** χ^2^ test of independence between patterning modes of the variegated pattern and pollination type in 34 Ligeriinae species.

**Pollination type**	**Variegated pattern**			**χ^2^**	***P*-value**
	**Random mode**	**Vascular mode**	**None**		
Bat	1	0	0	16.70	1.05 × 10^–2^
Bee	3	5	1		
Hummingbird	0	15	5		
Moth	0	0	1		

**TABLE 4:** χ^2^ test of independence between patterning modes of the gradient pattern and pollination type in 34 Ligeriinae species.

**Pollination type**	**Gradient pattern**			**χ^2^**	***P*-value**
	**Distal mode**	**Proximal mode**	**None**		
Bat	0	0	1	22.75	8.85 × 10^–4^
Bee	2	4	3		
Hummingbird	18	0	2		
Moth	0	0	1		

### Quantitative Nectar Guide Patterns and Their Association With the Pollination Type

**FIGURE 5:**
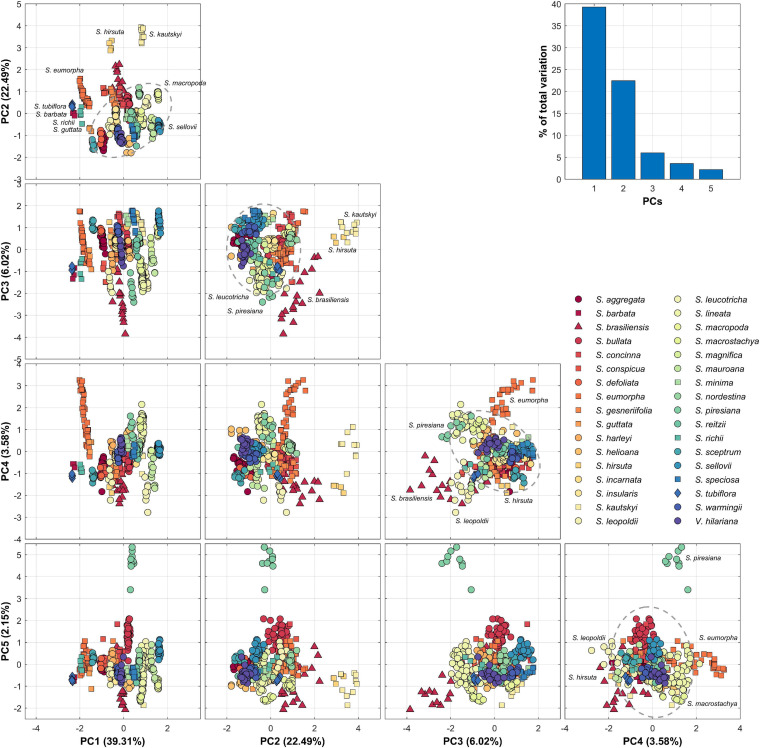
Summary and scatter plots of PC1–5. PC scores are standardized to a mean of 0 and variance of 1. Circle, triangle, square, and diamond represent hummingbird, bat, bee, and moth pollination types, respectively. The dashed-line ellipse denotes the confidence ellipse of hummingbird pollination type.

Nectar guide patterns were quantified by applying PCA to all 454 homologous ROIs. The first five PCs, namely PC1–PC5, accounted for 39.31, 22.49, 6.02, 3.58, and 2.15% of the total variance, respectively (Figure 5). In PC1–PC2 scatter plot, most of the species exhibiting bat, bee, and moth pollination types were distant from most of the species with the hummingbird pollination type. In PC3–PC4 and PC4–PC5 scatter plots, some species exhibiting the hummingbird pollination type, such as *Sinningia leopoldii* and *Sinningia piresiana*, with unique nectar guide patterns were also distant from other species exhibiting the hummingbird pollination type.

**FIGURE 6:**
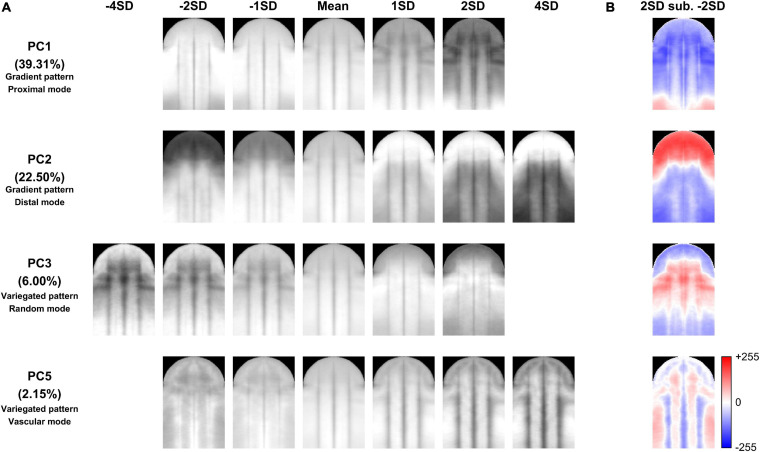
Illustration of the virtual homologous ROIs of PC1, PC2, PC3, and PC5 **(A)** and the deviation between the PC scores of the mean + 2 SDs and the mean – 2 SDs **(B)**.

The quantified nectar guide pattern accounting for the pollination type coincided with the patterning modes defined in the qualitative analysis. The variation in PC1, PC2, PC3, and PC5 significantly explained the variation in pollination types in the stepwise multiple regression analysis (Table 5, *P* = 1.8187 × 10^–70^). Figure 6A illustrates the virtual homologous ROIs of PC1, PC2, PC3, and PC5 with the PC scores (means ± standard deviations, SDs) covering negative and positive extremes. Figure 6B shows the deviation between the PC scores of the mean + 2 SDs and the mean – 2 SD. By determining the location of the larger gray-level deviation, we found that the variation in PC1, PC2, PC3, and PC5 corresponded to the proximal, distal, random, and vascular modes, respectively.

**TABLE 5:** Stepwise multiple regression analysis for PCs predicting pollination types.

	**Stepwise multiple regression analysis**							
	***R*^2^ (%)**	***F*-stat^*a*^**	***P*-value**	**B^*b*^**	**SE^*b*^**	***T*-stat^*b*^**	***P*-value**	**Status^*c*^**
	51.97	121.98	1.82 × 10^–70^					
PC1				–0.65	0.04	−15.27	9.84 × 10^–43^	In
PC2				0.66	0.04	15.49	1.03 × 10^–43^	In
PC3				0.097	0.04	2.27	2.35 × 10^–2^	In
PC4				0.082	0.04	1.93	5.48 × 10^–2^	Out
PC5				–0.13	0.04	−3.09	2.10 × 10^–3^	In

*^*a*^*F*-stat, the *F*-statistic of testing the final model versus the initial model (empty variable). ^*b*^B, coefficient for variables in the final model; SE, the standard error of coefficient estimates; *T*-stat, the *T-*statistic of coefficient estimation. ^*c*^Status, the decision for variables to be included in the model. The tolerance level for adding and removing variables is 0.05.*

The quantified nectar guide pattern showed a strong association with pollination type. In the association analysis, species exhibiting bee and hummingbird pollination types were only included because only one species each demonstrated bat and moth pollination types. The LOD score and permutation test results revealed that PC1 (LOD = 19.91, *P* = 1.74 × 10^–31^), PC2 (LOD = 30.57, *P* = 1.36 × 10^–36^), and PC5 (LOD = 0.70, *P* = 4.95 × 10^–2^) were significantly associated with bee and hummingbird pollination types at a significance level of 0.05 (Table 6). The high LOD scores of PC1 and PC2 coincided with the result in the qualitative analysis that the gradient pattern had a stronger association with pollination type than that of the variegated pattern and pollination type.

**TABLE 6:** Logarithm of the odds scores of PC1, PC2, PC3, and PC5 for hummingbird and bee pollination types.

	**LOD score**	***P*-value**
PC1 (proximal mode of gradient pattern)	19.91	1.74 × 10^–31^
PC2 (distal mode of gradient pattern)	30.57	1.36 × 10^–36^
PC3 (random mode of variegated pattern)	0.56	9.19 × 10^–2^
PC5 (vascular mode of variegated pattern)	0.70	4.95 × 10^–2^

## Discussion

In this study, we proposed defining a homologous ROI to compare nectar guide patterns on the developmentally homologous regions between species with different petal shapes and sizes. By using homologous ROIs, we qualified and quantified nectar guide patterns. In addition, we provided statistical evidence suggesting the association of nectar guide patterns with pollination types.

### More Patterning Modes to Be Identified Using Homologous ROIs

In Ligeriinae, some unique patterning modes apart from the four identified patterning modes, remain to be defined and identified. The proposed homologous ROI only acquired the middle vein and two side veins to represent the petal vasculature. In *Sinningia bullata*, pigmentation following the higher-order vein was evident (Figure 1), forming strips that were not parallel to the middle vein and two side veins in the homologous ROI (Figure 4). This observation implied that the vascular mode might be too coarse to describe the diversity of pigmentation patterning related to the vasculature. Regarding the higher-order vein, we also found a patterning mode contrasting with the vascular mode. In *S. conspicua*, at the region between the middle and side veins, the pigmented area evaded the vasculature of higher-order veins. In addition to the patterning modes of the variegated pattern, an undescribed patterning mode of the gradient pattern exists. In *Sinningia minima*, the process of pigmentation emerging from the neighboring lateral tube toward the side vein of the ventral tube was observed in the developmental series. Thus, an unpigmented area formed at the center of the ventral petal. The homologous ROI remains to be further refined to sufficiently and appropriately identify variations in nectar guide patterns.

### Insights Into the Pollination Type Based on the Patterning Mode of Nectar Guide Patterns

The results of our study indicate that the color distribution of petals, namely the nectar guide pattern, was strongly associated with different types of pollinators. A recent study reported the effect of petal color on pollinator preference in the neotropical Gesneriaceae (Ogutcen et al., 2021). Our study results imply that color distribution is also linked to the innate preference of pollinators and the pollination strategy in plants. Studies examining the pattern preferences of bees have shown that the color contrast between the inner and outer corolla (Lunau et al., 1996, 2006), the color patch at the center of the corolla (Free, 1970), and the pattern of dotted lines and stripes (radiating sector; Lehrer et al., 1995; Leonard and Papaj, 2011) can effectively allure bees. Our results coincide with those of these studies. A large proportion of species exhibiting the bee pollination type recruited vascular and proximal modes. Furthermore, most of the species exhibiting the hummingbird pollination type recruited vascular and distal modes. This finding also coincides with that a previous study demonstrating that long corolla tubes with color contrast is conspicuous to hummingbirds (Bergamo et al., 2019). *Sinningia tubiflora*, the only species that exhibited the moth pollination type, lacked variegated and gradient patterns, suggesting that visual attraction is not its major strategy for attracting moths (Knudsen and Tollsten, 1993). Similarly, acoustic and scent attraction, rather than visual attraction, are major strategies for species demonstrating the bat pollination type (Gonzalez-Terrazas et al., 2016). This may explain why only *S. brasiliensis* has green petals and demonstrated a highly fluctuating random mode among specimens (Supplementary Figure 2). In addition, we also added more species in the analysis, which included the species with petal images captured under undesirable conditions (Supplementary Figure 3) and the species from the literature and image galleries on the Internet (Supplementary Table 1). We found that the association between patterning modes and pollination types was stronger and obvious (Supplementary Tables 2, 3). The present study provides insights into the association of patterning modes with pollination types from the perspective of nectar guide pattern diversity.

### Application and Generalization of Homologous ROIs

The extraction of homologous ROIs integrates plant histology and image processing and provides new insights into the study of nectar guide patterns. Homologous ROIs can be applied to quantitative genetics. PC scores can serve as trait values and present trait differences between a parent and its offspring. Supplementary Figure 4 presents the quantified nectar guide patterns of F_1_ hybrids. With the use of homologous ROIs, the interspecific comparative analysis can also be expanded. Supplementary Figure 5 shows an example in which homologous ROIs are applied to Gesneriaceae species other than Ligeriinae. PC scores can also serve as character values for phylogenetic analysis. Supplementary Figure 6 summarizes the test of phylogenetic signals indicating the nectar guide pattern of the related species have less tendency to resemble each other in Ligeriinae.

Biologists can use homologous ROIs to gain a comprehensive understanding of nectar guide patterns with an extensive survey. In Ronse De Craene’s (2008) study, the petaloid organ vasculature of Lamiales (including the family Gesneriaceae) belongs to the single bundle character state. Approximately 70% of the taxa of eudicots was determined to have this character state. The single bundle was further subdivided into fan-like and trifid branching types. Although Lamiales was identified to have the fan-like branching type, the vasculature of the single middle vein and two side veins was clearly recognized in the present study. These findings suggest that the homologous ROI has potential to be generalized to the taxa adopting a single bundle development of the petal vasculature. As aforementioned, the petal is a homologous structure across core eudicots. Thus, homologous ROIs may be applied to other Lamiales lineages, or even the core eudicot, to perform a large-scale comparative analysis of nectar guide patterns.

## Data Availability Statement

The raw data supporting the conclusions of this article will be made available by the authors, without undue reservation.

## Author Contributions

H-CH: conceptualization, methodology, software, validation, formal analysis, investigation, resources, data curation, writing – reviewing and editing, visualization, and project administration. Y-FK: conceptualization, resources, writing – reviewing and editing, supervision, project administration, and funding acquisition. Both authors contributed to the article and approved the submitted version.

## Conflict of Interest

The authors declare that the research was conducted in the absence of any commercial or financial relationships that could be construed as a potential conflict of interest.

## Supplementary Material

Supplementary Figure 1 Developmental serial of *Sinningia brasiliensis* (K024074), *Sinningia eumorpha* (K039133), *Sinningia magnifica* (K039166), *Sinningia nordestina* (K039168), *Sinningia sceptrum* (K039182), and *Sinningia speciosa* (K039190). V_*R*_: random mode of variegated pattern; V_*V*_: vascular mode of variegated pattern; G_*D*_: distal mode of gradient pattern; G_*P*_: proximal mode of gradient pattern.

Supplementary Figure 2 Homologous ROI of *Sinningia brasiliensis*.

Supplementary Figure 3 Homologous ROI of *Sinningia aghensis*, *Sinningia allagophylla*, *Sinningia carangolensis*, *Sinningia polyantha*, and *Sinningia pusilla*. Scale bar: 0.5 cm.

Supplementary Figure 4 Ventral petals, homologous ROIs, and quantified nectar guide patterns in the PC1 and PC2 of eight F_1_ hybrids. Scale bar: 0.5 cm.

Supplementary Figure 5 Ventral petals, homologous ROIs, and quantified nectar guide patterns in the PC1 and PC2 of eight Gesneriaceae species. Scale bar: 0.5 cm.

Supplementary Figure 6 Phylogenetic signals of 4 PC scores. The boxplot presents the distributions of the Blomberg K values obtained from the phylogenetic trees of 1,000 replicates in the maximum likelihood analysis. Diamonds indicate the Blomberg K values calculated using the 50% majority-rule consensus tree. The gray dots in the denote the data point of outliers. The *P*-values are provided at the right of each boxplot.

Supplementary Table 1 Qualitative nectar guide pattern of 60 Ligeriinae species reviewed from the literature and image galleries on the Internet.

Supplementary Table 2 χ^2^ test of independence between the patterning modes of the variegated pattern and pollination type in 83 Ligeriinae species.

Supplementary Table 3 χ^2^ test of independence between the patterning modes of the gradient pattern and pollination type in 83 Ligeriinae species.
