# Supplementary material for: Nectar Guide Patterns on Developmentally Homologous Regions of the Subtribe Ligeriinae (Gesneriaceae)
Source: Front Plant Sci. 2021 Apr 12;12:650836. doi: 10.3389/fpls.2021.650836 (PMC8072120; doi:10.3389/fpls.2021.650836)
Supplement: Supplementary Figure 1 — Developmental serial of Sinningia brasiliensis (K024074), Sinningia eumorpha (K039133), Sinningia magnifica (K039166), Sinningia nordestina (K039168), Sinningia sceptrum (K039182), and Sinningia speciosa (K039190). VR: random mode of variegated pattern; VV: vascular mode of variegated pattern; GD: distal mode of gradient pattern; GP: proximal mode of gradient pattern. [file Data_Sheet_1.PDF]

Supplementary Material

1 Supplementary Figures

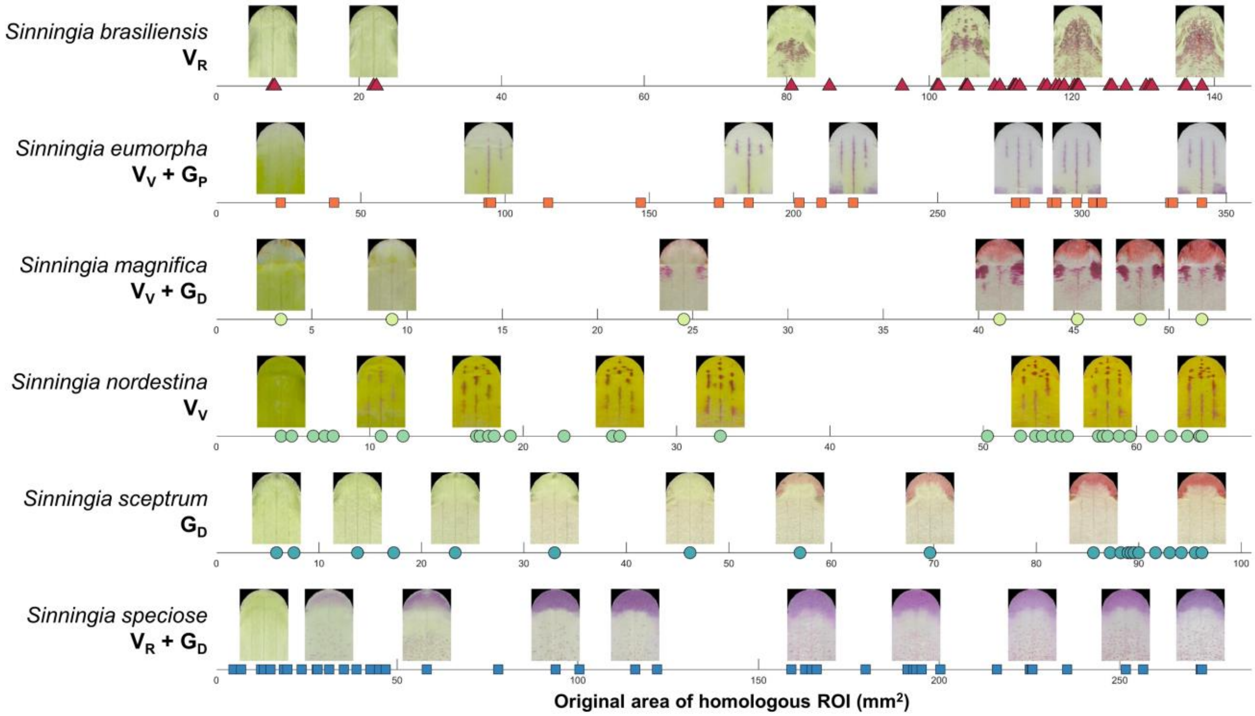

Supplementary Figure 1: Developmental serial of *Sinningia brasiliensis* (K024074), *Sinningia eumorpha* (K039133), *Sinningia magnifica* (K039166), *Sinningia nordestina* (K039168), *Sinningia sceptrum* (K039182), and *Sinningia speciose* (K039190). V_R: random mode of variegated pattern; V_V: vascular mode of variegated pattern; G_D: distal mode of gradient pattern; G_P: proximal mode of gradient pattern.

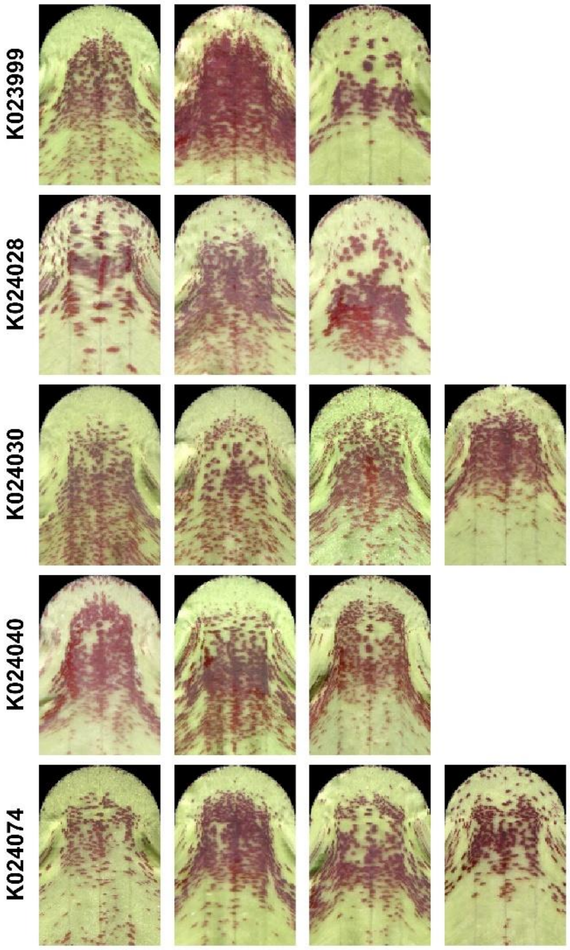

Supplementary Figure 2: Homologous ROI of *Sinningia brasiliensis*.

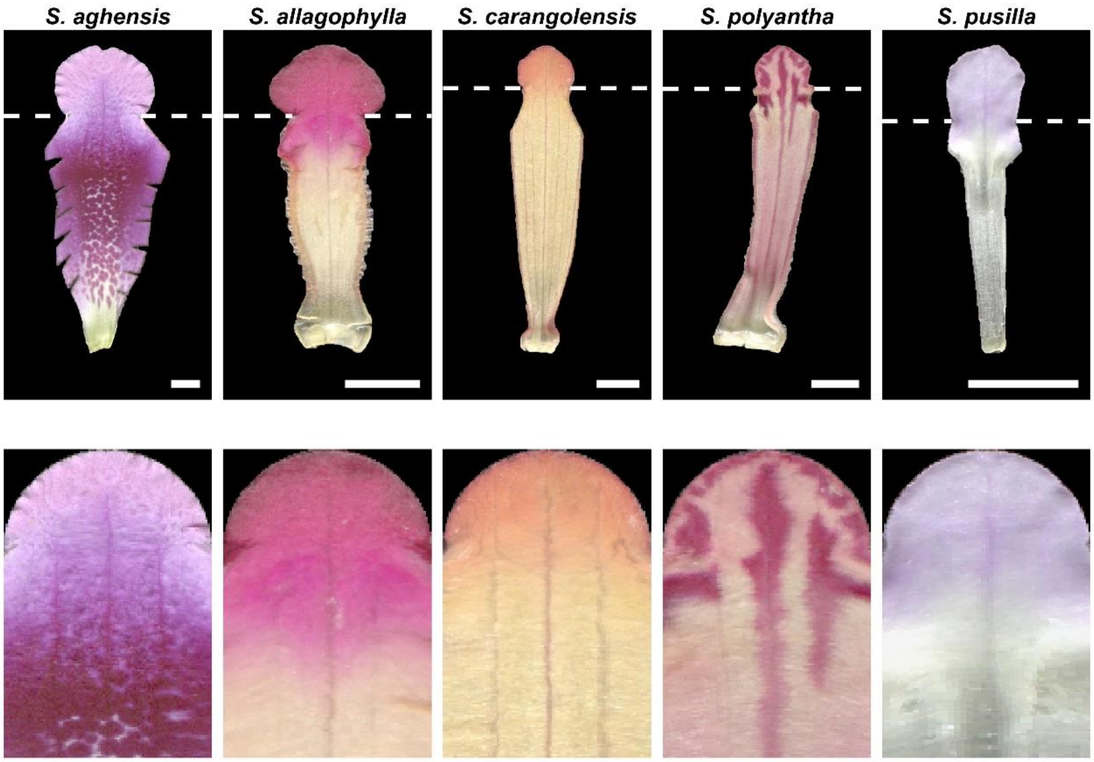

Supplementary Figure 3: Homologous ROI of *Sinningia aghensis*, *Sinningia allagophylla*, *Sinningia carangolensis*, *Sinningia polyantha*, and *Sinningia pusilla*. Scale bar: 0.5 cm.

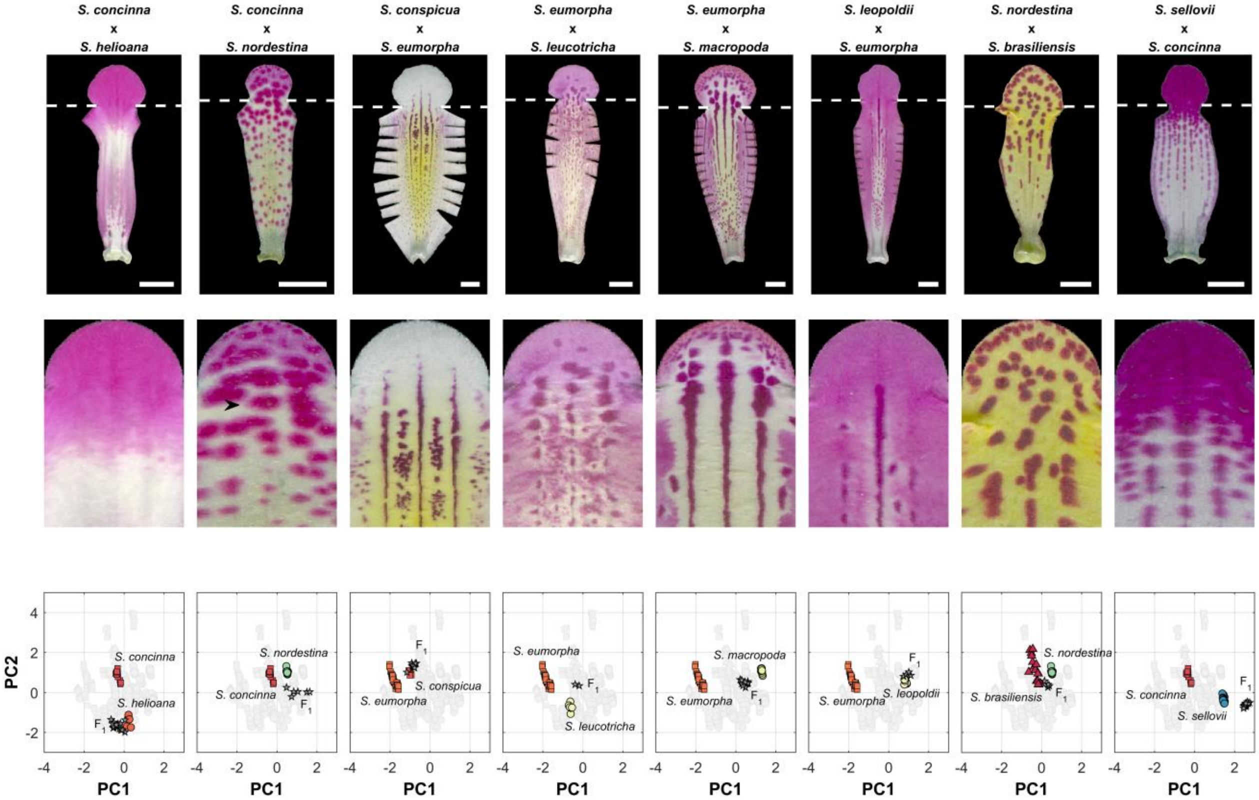

Supplementary Figure 4: Ventral petals, homologous ROIs, and quantified nectar guide patterns in the PC1 and PC2 of eight F_1 hybrids. Scale bar: 0.5 cm.

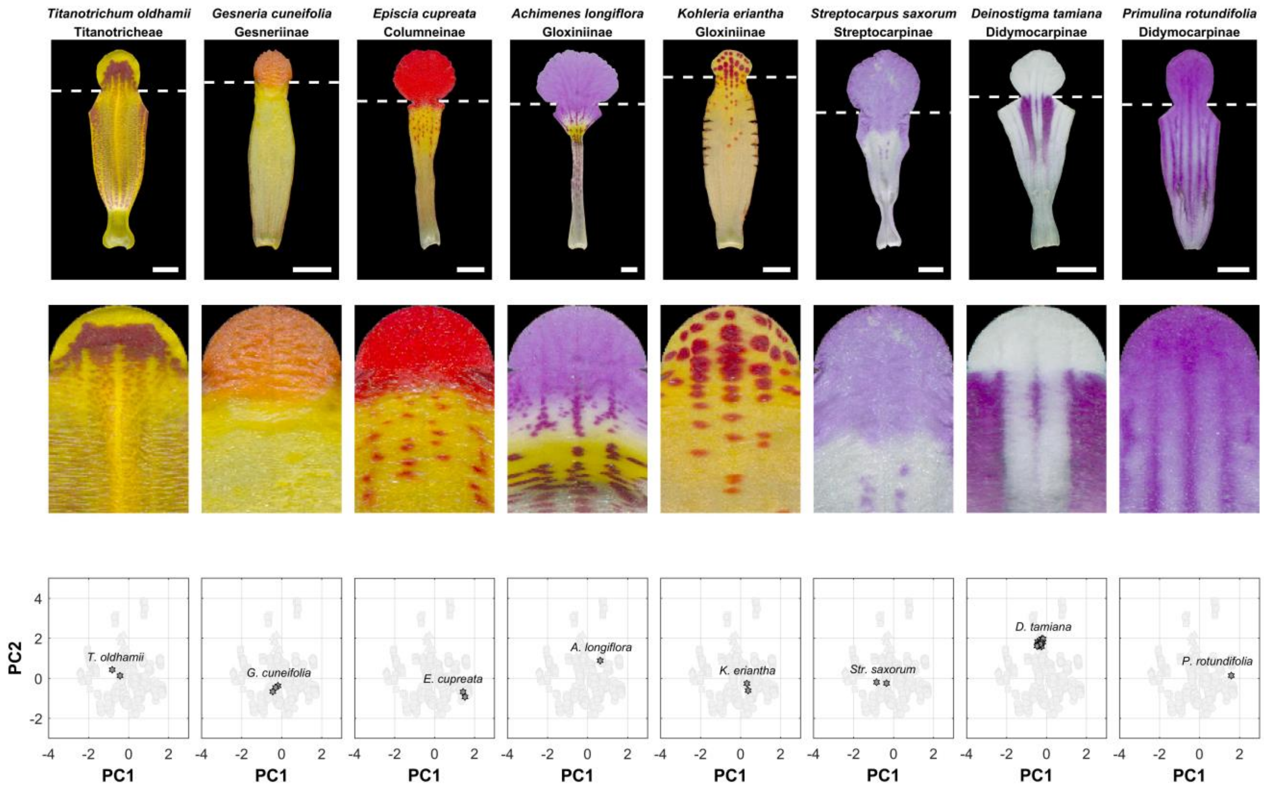

Supplementary Figure 5: Ventral petals, homologous ROIs, and quantified nectar guide patterns in the PC1 and PC2 of eight Gesneriaceae species. Scale bar: 0.5 cm.

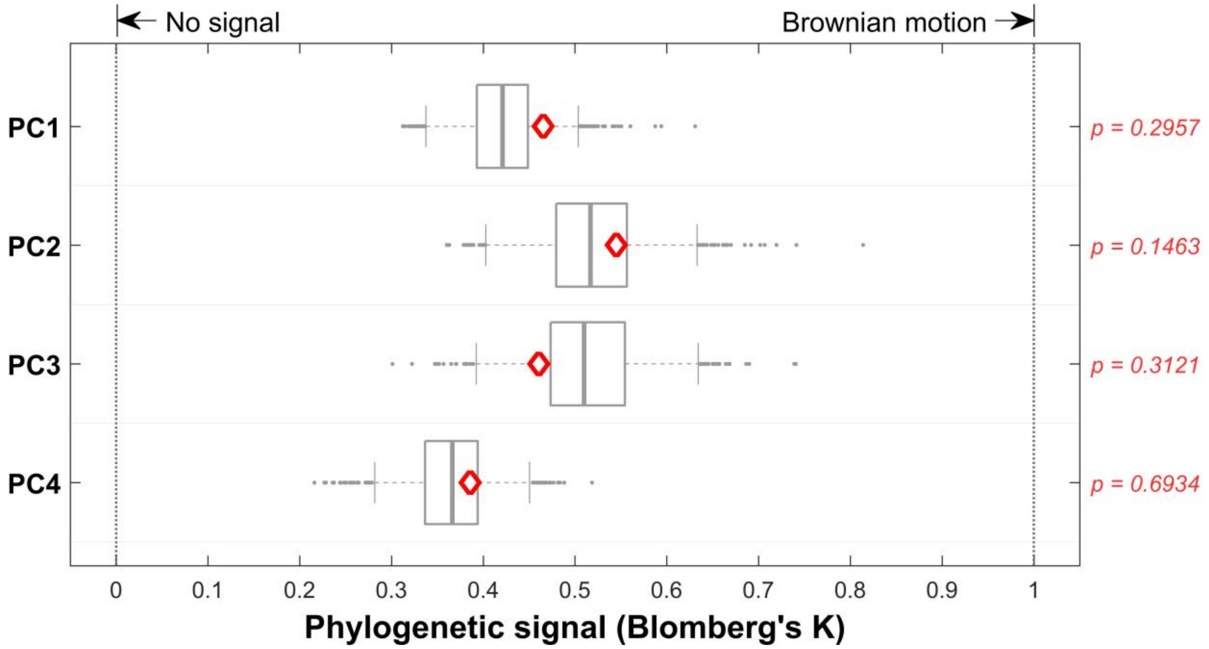

Supplementary Figure 6: Phylogenetic signals of 4 PC scores. The boxplot presents the distributions of the Blomberg K values obtained from the phylogenetic trees of 1,000 replicates in the maximum likelihood analysis. Diamonds indicate the Blomberg K values calculated using the 50% majority-rule consensus tree. The gray dots in the denote the data point of outliers. The *P*-values are provided at the right of each boxplot.

2 Supplementary Tables

Supplementary Table 1: Qualitative nectar guide pattern of 60 Ligeriinae species reviewed from the literature and image galleries on the Internet.

Taxa [a]	Pollination type (Perret et al, 2003)	Variegated pattern (**R**andom v.s. **V**ascular-dependent)	Gradient pattern (**D**istal v.s. **P**roximal)	Sources of photos and figures
Paliavana gracilis	Bee	V	D	Peixoto and Pereira (2020)
Paliavana plumerioides	Bee	weak R	--	Peixoto and Pereira (2020)
Paliavana prasinata	Bat	--	--	Grice (2020); LaVergne (2020); Peixoto and Pereira (2020); Sanmartin-Gajardo and Sazima (2005)
Paliavana sericiflora	Bat/Hummingbird [b]	R (some varieties V) [f]	--	Peixoto and Pereira (2020); Sanmartin-Gajardo and Sazima (2005)
Paliavana tenuiflora	Bee	R	P	Ferreira et al. (2010); Grice (2020); Peixoto and Pereira (2020); Sales (2015)
Paliavana werdermannii	Bat	weak V	--	Peixoto and Pereira (2020)
Sinningia aghensis	Bee	R	P	Grice (2020); Peixoto and Pereira (2020); Fig. S3 of the present study
Sinningia allagophylla	Hummingbird	--	D	Ferreira et al. (2015); Peixoto and Pereira (2020); Fig. S3 of the present study Ferreira et al. (2015)
Sinningia amambayensis	Hummingbird	V	D	LaVergne (2020)
Sinningia araneosa	Hummingbird	weak V	D	Grice (2020); LaVergne (2020)
Sinningia bragae	Bee [c]	weak R	P	Chautems et al. (2015); LaVergne (2020)
Sinningia bulbosa	<u>*to be determined*</u>	<u>*to be determined*</u>	<u>*to be determined*</u>	
Sinningia calcaria	Hummingbird	weak V	D	Grice (2020); LaVergne (2020)
Sinningia canastrensis	Bee	V	D	Chautems et al. (2010); Peixoto and Pereira (2020); Sanmartin-Gajardo and Sazima (2005)
Sinningia canescens	Hummingbird	V	D	Grice (2020); Peixoto and Pereira (2020)
Sinningia carangolensis	Hummingbird	--	D	Peixoto and Pereira (2020); Fig. S3 of the present study
Sinningia cardinalis	Hummingbird	V	D	LaVergne (2020); Peixoto and Pereira (2020); Winefield et al. (2005)
Sinningia cochlearis	Hummingbird	--	D	LaVergne (2020); Peixoto and Pereira (2020)
Sinningia cooperi	Hummingbird	R	D	Blaser et al. (2012)
Sinningia curtiflora	Hummingbird	--	D	Ferreira et al. (2015); Peixoto and Pereira (2020)
Sinningia douglasii	Hummingbird	V	D	Ferreira et al. (2015); Peixoto and Pereira (2020)
Sinningia elatior	Hummingbird	--	D	Araújo and Rocha-Filho (2019); Ferreira et al. (2015); Peixoto and Pereira (2020)
Sinningia flammea	Hummingbird [d]	--	D	Chautems et al. (2019); LaVergne (2020); Peixoto and Pereira (2020)
Sinningia gerdtiana	Bee	V	--	Chautems et al. (2010); Dutra (2018); LaVergne (2020); Peixoto and Pereira (2020)
Sinningia gigantifolia	Hummingbird	--	D	Arzolla et al. (2007); Peixoto and Pereira (2020)
Sinningia glazioviana	Hummingbird	V	D	LaVergne (2020)
Sinningia globulosa	Hummingbird	V	D	Chautems et al. (2010); LaVergne (2020)
Sinningia hatschbachii	Hummingbird	weak V	D	Grice (2020); LaVergne (2020) ; Peixoto and Pereira (2020)

Sinningia helleri	Bee	V	P	Dutra (2018); LaVergne (2020); Peixoto and Pereira (2020)
Sinningia hoehnei	Bee [d]	V	P	Chautems et al. (2019); LaVergne (2020); Peixoto and Pereira (2020)
Sinningia iarae	Hummingbird	weak V	D	LaVergne (2020); Peixoto and Pereira (2020)
Sinningia lateritia	Hummingbird	*to be determined*	*to be determined*	
Sinningia lindleyi	Bee	V	P	Grice (2020); LaVergne (2020); Peixoto and Pereira (2020)
Sinningia lutea	Hummingbird [e]	--	--	Buzatto and Singer (2012)
Sinningia micans	*to be determined*	*to be determined*	*to be determined*	
Sinningia musicola	Bee	--	D	Chautems et al. (2010); Grice (2020)
Sinningia nivalis	Hummingbird	V	D	Ferreira et al. (2015); Grice (2020)
Sinningia polyantha	Hummingbird	V	D	Dutra (2018); LaVergne (2020); Peixoto and Pereira (2020); Fig. S3 of the present study
Sinningia punctata	*to be determined*	*to be determined*	*to be determined*	
Sinningia pusilla	Bee	weak V	D	Dutra (2018); Grice (2020); Peixoto and Pereira (2020); Fig. S3 of the present study
Sinningia ramboi	Hummingbird	V	D	Ferreira et al. (2015); Grice (2020); LaVergne (2020); Peixoto and Pereira (2020)
Sinningia rupicola	Hummingbird	V	D	Grice (2020); Peixoto and Pereira (2020)
Sinningia schiffneri	Bee	R	--	Grice (2020); LaVergne (2020); Peixoto and Pereira (2020); Sanmartin-Gajardo and Sazima (2005)
Sinningia schomburgkiana	*to be determined*	*to be determined*	*to be determined*	
Sinningia stapelioides	Hummingbird [d]	V	weak D	Chautems et al. (2019); Grice (2020); LaVergne (2020); Peixoto and Pereira (2020)
Sinningia striata	Hummingbird	V	D	LaVergne (2020); Peixoto and Pereira (2020)
Sinningia sulcata	Hummingbird	--	--	LaVergne (2020); Peixoto and Pereira (2020)
Sinningia tuberosa	Hummingbird	V	D	Blaser et al. (2012); Grice (2020)
Sinningia valsuganensis	Hummingbird	V	D	LaVergne (2020); Peixoto and Pereira (2020)
Sinningia velutina	Bee	V	--	Peixoto and Pereira (2020)
Sinningia villosa	Bee	V	P	Grice (2020); Sanmartin-Gajardo and Sazima (2005); Peixoto and Pereira (2020)
Vanhouttea bradeana	*to be determined*	*to be determined*	*to be determined*	
Vanhouttea brueggeri	Hummingbird	V	D	Blaser et al. (2012); Chautems (2002); LaVergne (2020); Peixoto and Pereira (2020)
Vanhouttea calcarata	Hummingbird	V	D	LaVergne (2020); Peixoto and Pereira (2020)
Vanhouttea fruticulosa	Hummingbird	--	D	Peixoto and Pereira (2020)
Vanhouttea gardneri	*to be determined*	*to be determined*	*to be determined*	
Vanhouttea lanata	Hummingbird	V	D	Grice (2020); LaVergne (2020); Peixoto and Pereira (2020)
Vanhouttea leonii	Hummingbird	V	D	LaVergne (2020)
Vanhouttea mollis	*to be determined*	*to be determined*	*to be determined*	
Vanhouttea pendula	Hummingbird	V	D	LaVergne (2020)

[a] The list of taxa was delivered from the International Plant Names Index and the Plants of the World Online.

[b] The hummingbird pollination was confirmed by Sanmartin-Gajardo and Sazima (2005).

[c] The pollination type was referenced from Chautems et al. (2015). The pollination type of *S. bragae* was inferred from the corolla morphology of *S. aghensis*.

[d] The pollination types were referenced from Chautems et al. (2019). The pollination type of *S. flammea* was inferred from the corolla morphology of *S. helioana*; S. *hoehnei* from *S. barbata*.

[e] The pollination type was referenced from Buzatto and Singer (2012). The pollination type of *S. lutea* was inferred from the corolla morphology of *S. allagophylla*.

[f] In the chi-square test of independence, the species was duplicated. One is the random mode and the other one is the vascular mode.

Araújo, F. P. D., & Rocha-Filho, L. C. D. (2019). Special offer while stocks last: high-energy pulse of nectar caused by a massive post-fire flowering of Sinningia elatior (Gesneriaceae) in an area of Cerrado and implications for its pollination. *Rodriguésia*, 70.

Arzolla, F. A. R. D. P., Paula, G. C. R. D., Chautems, A. P., & Shepherd, G. J. (2007). O primeiro registro de Sinningia gigantifolia Chautems (Gesneriaceae) no estado de São Paulo. *Biota Neotropica*, *7*(3), 373-377.

Blaser, J. G., Salimena, F. R. G., & Chautems, A. (2012). Gesneriaceae of Serra Negra, Minas Gerais, Brazil. *Rodriguésia*, *63*(3), 705-714.

Buzatto, C. R., & Singer, R. B. (2012). Sinningia lutea (Gesneriaceae), a new species from Southern Brazil. *Brittonia*, 64(2), 108-113.

Chautems, A. (2002). New Gesneriaceae from Minas Gerais, Brazil. *Candollea*, 56(2), 261-279.

Chautems, A., Dutra, V. F., Fontana, A. P., Peixoto, M., Perret, M., & Rossini, J. (2019). Three new species of Sinningia (Gesneriaceae) endemic to Espírito Santo, Brazil. *Candollea*, 74(1), 33-42.

Chautems, A., Lopes, T. C. C., Peixoto, M., & Rossini, J. (2010). Taxonomic revision of Sinningia Nees (Gesneriaceae) IV: six new species from Brazil and a long overlooked taxon. *Candollea*, 65(2), 241-266.

Chautems, A., Peixoto, M., & Rossini, J. (2015). A new species of Sinningia Nees (Gesneriaceae) from Espírito Santo and Rio de Janeiro states, Brazil. *Candollea*, 70(2), 231-235.

Dutra, F. V. (2018). *Palinotaxonomia de espécies brasileiras de Sinningia Nees (Gesneriaceae)-evolução da morfologia polínica e síndromes de polinização* (Doctoral dissertation, Universidade de São Paulo).

Ferreira, G. E., Chautems, A., & Waechter, J. L. (2015). Taxonomy of Sinningia Nees (Gesneriaceae) in Rio Grande do Sul, southern Brazil. *Acta Botanica Brasilica*, 29(3), 310-326.

Ferreira, P., Horn, M., & Glemser, E. (2010). Floral visitors of Paliavana tenuiflora (Mansf.) (Gesneriacae) in Mucugê, Bahia, Brazil. *Biologia e Ecologia da Polinização*, 33.

Grice, M. (2020, Jul 1). The Gesneriad Society. Retrieved from https://www.gesneriadsociety.org/

LaVergne, A. (2020, Jun 1). Sinningia & Friends. Retrieved from http://www.burwur.net/sinns/sinns.htm

Peixoto, M; Pereira, S. A. (2020, Feb 25). BRAZIL PLANTS. Retrieved from http://www.brazilplants.com/

Sales, K. A. (2015). *Constituintes químicos de Paliavana tenuiflora Mansf.(Gesneriaceae)*.

SanMartin-Gajardo, I., & Sazima, M. (2004). Non-euglossine bees also function as pollinators of Sinningia species (Gesneriaceae) in southeastern Brazil. *Plant Biology*, *6*(4), 506-512.

Sanmartin-Gajardo, I., & Sazima, M. (2005). Chiropterophily in Sinningieae (Gesneriaceae): *Sinningia brasiliensis* and *Paliavana prasinata* are bat-pollinated, but *P. sericiflora* is not. Not yet?. *Annals of Botany*, *95*(7), 1097-1103.

Wiehler, H. (1984). Miscellaneous new species in the Gesneriaceae. *Selbyana*, 7(2/4), 328-347.

Winefield, C. S., Lewis, D. H., Swinny, E. E., Zhang, H., Arathoon, H. S., Fischer, T. C., ... & Davies, K. M. (2005). Investigation of the biosynthesis of 3-deoxyanthocyanins in Sinningia cardinalis. *Physiologia Plantarum*, 124(4), 419-430.

Supplementary Table 2: χ^2 test of independence between the patterning modes of the variegated pattern and pollination type in 83 Ligeriinae species.

Pollination type	Variegated pattern			Chi-square value	*p*-value
	**R**andom mode	**V**ascular mode	**N**one		
Bat	2	2	1	22.62	9.33×10^{-4}
Bee	8	14	2		
Hummingbird	1	38	15		
Moth	0	0	1		

Supplementary Table 3: χ^2 test of independence between the patterning modes of the gradient pattern and pollination type in 83 Ligeriinae species.

Pollination type	Gradient pattern			Chi-square value	*p*-value
	**D**istal mode	**P**roximal mode	**N**one		
Bat	0	0	4	63.95	7.07×10^{-12}
Bee	6	11	7		
Hummingbird	50	0	4		
Moth	0	0	1		
